# Replacement of hematopoietic system by allogeneic stem cell transplantation in myelofibrosis patients induces rapid regression of bone marrow fibrosis

**DOI:** 10.1186/1755-1536-5-S1-S25

**Published:** 2012-06-06

**Authors:** Nicolaus Kröger, Michael Kvasnicka, Jürgen Thiele

**Affiliations:** 1 Dept. of Stem Cell Transplantation, University Hospital Hamburg, Germany; 2 Institute for Pathology, University Hospital Cologne, Germany

## Abstract

Bone marrow fibrosis is a hallmark of primary and post ET/PV myelofibrosis. To investigated the impact of replacement of the hematopoietic system in myelofibrosis patients by allogeneic stem cell transplantation on bone marrow fibrosis, we studied bone marrow fibrosis on bone marrow samples from 24 patients with myelofibrosis before and after dose-reduced conditioning followed by allogeneic stem cell transplantation from related or unrelated donor. Using the European Consensus on Grading Bone Marrow Fibrosis, before allografting all patients had advanced fibrosis MF-2 (n = 13) or MF-3 (n = 11). After transplantation, a complete (MF-0) or nearly complete (MF-1) regression of bone marrow fibrosis was seen in 59 % at day +100, in 90 % at day +180, and in 100 % at day +360. No correlation between occurrence of acute graft-versus-host disease, and fibrosis regression on day +180 was seen. We conclude that dose-reduced conditioning, followed by allogeneic stem cell transplantation, resulted in a rapid resolution of bone-marrow fibrosis suggesting the bone marrow fibrogenesis is a highly dynamic rather than static process in patients with myelofibrosis.

## Introduction

Myelofibrosis with myeloid metaplasia (MMM) is a rare malignant hematological disease of the hematopoietic stem cells with a median age at diagnosis of 60 years. Myelofibrosis is characterized by splenomegaly, extramedullary hematopoiesis, bone marrow collagen fibrosis, and osteosclerosis [1]. Bone marrow fibroblasts in patients with myelofibrosis are polyclonal and exhibit normal function [2]. Clinical and preclinical observation suggested that cellular and extracellular levels of fibrogenic and angiogenic cytokines are altered in patients with myelofibrosis Conventional therapy is mainly used to control symptoms whereas no such therapy has so far shown to change the natural course of the disease. The only curative treatment option is hematopoietic stem cell transplantation [3]. With allogeneic stem cell transplantation, about 50 % of the patients can be cured from their disease but the effect on bone marrow fibrosis has not been investigated systematically so far. Therefore we evaluated bone-marrow-fibrosis regression in patients with myelofibrosis after dose-reduced conditioning (busulfan, 10 mg/kg BW; fludarabine, 180 mg/m²; and ATG, 30 - 60 mg/kg BW), followed by allogeneic stem cell transplantation from related (n = 6) or un-related (n = 18) donor.

## Methods and patients

Twenty-four patients (male: n = 16; female: n = 8) with a median age of 52 years (range, 32-63) were included. There were 16 male and 8 female patients. Diagnosis was primary myelofibrosis in 18 patients and secondary myelofibrosis in six patients; four of them developed from polycythaemia vera, and two of them from essential thrombocythaemia. Using the European Consensus on Grading [4]. This consensus distinguishes between:

MF - 0 Scattered linear reticulin with no intersections [cross-overs] corresponding to normal bone marrow

MF - 1 Loose network of reticulin with many intersections, especially in paravascular areas

MF - 2 Diffuse and dense increase in reticulin with extensive intersections, occasionally with only focal bundles of collagen and/or focal osteosclerosis

MF - 3 Diffuse and dense increase in reticulin with extensive intersections with coarse bundles of collagen, often associated with significant osteosclerosis

Fibrosis grading was determined independently by two experienced hemato-pathologists.

All patients underwent bone marrow biopsy prior allogeneic stem cell transplantation. A first biopsy after transplantation should be performed between day +30 and day +100, a second bone marrow histology on day +180, and a third histology one year after transplantation. Patients could be included if they had at least one bone marrow histology within the first 180 days after transplantation. The study was approved by the ethic committee of Hamburg/Germany. All patients gave written informed consent for participation in the study.

## Results

From all patients, bone marrow histology was obtained before allogeneic stem cell transplantation (n = 24). After transplantation, 76 bone marrow histologies were performed. Bone Marrow Fibrosis, before allografting all patients had advanced fibrosis MF-2 (n = 13) or MF-3 (n = 11). After transplantation, a complete (MF-0) or nearly complete (MF-1) regression of bone marrow fibrosis was seen in 59 % at day +100, in 90 % at day +180, and in 100 % at day +360. (see table 1). No correlation between occurrence of acute graft-versus-host disease, and fibrosis regression on day +180 was seen.

**Table 1:** 

Grade*	n = 24	n = 22	n= 20	n = 16
	
	prior allograft	day +30 to day +100	day +180	day +360
**3**	11 (49 %)	2 (9 %)	0	0
**2**	13 (51 %)	7 (32 %)	2 (10 %)	0
**1**	0	9 (41 %))	9 (45 %)	6 (37 %)
**0**	0	4 (18 %)	9 (45 %)	10 (63 %)

^*^according to the European Consensus on Grading Bone Marrow Fibrosis

## Discussion

The pathogenesis of bone marrow fibrosis is not completely understood. A pathogenic role for developing bone marrow fibrosis is suggested for transforming-growth-factor beta (TGF-β), platelet-derived-growth factor (PDGF), basis-fibroblast-growth factor (BFGF), and vascular-endothelial-growth factor (VEGF) [2,5,6]. Furthermore also the interactions between cytokine release, myeloid stoma and other bone-marrow cells are only partially understood. Megakaryocytes are suggested to play a major role as source of cytokine release [6,7]. Furthermore, pathologic interaction between megakaryocytes and neutrophiles (emperipolesis) contributes to abnormal cytokine release [8]. Although the bone marrow texture is distored by fibrosis, donor-stem cells can engraft after a reduced intensity conditioning without obvious delay, suggesting that the bone marrow milieu for stem cell homing is apparently not seriously altered. It sounds reasonable that replacement of abnormal clonal hematopoietic cell population by allogeneic stem cell transplantation would eliminate the stimulus for abnormal cytokine release. Indeed several reports of allogeneic stem cell transplantation after standard conditioning for myelofibrosis reported a reversal of bone marrow fibrosis between six and 12 months after transplantation [9,10]. Since reduced intensity conditioning followed by allogeneic stem cell transplantation is becoming a reasonable treatment option for elder patients with inter-mediate or high-risk myelofibrosis [3], we systematically investigated the kinetics and dynamics of fibrosis regression in bone marrow after dose-reduced conditioning followed by allogeneic stem cell transplantation. Surprisingly, more than 59 % of the patients achieved complete or nearly complete regression of fibrosis (MF-1 and MF-0 shortly after engraftment at day +30; this percentage increased to 90 % at day +180, and to 100 % after one year. This rapid regression of bone marrow fibrosis suggests that fibrogenesis in myelofibrosis patients is a rather dynamic than static process.

## Competing interests

The authors declare that they have no competing interests.

**Figure 1:**
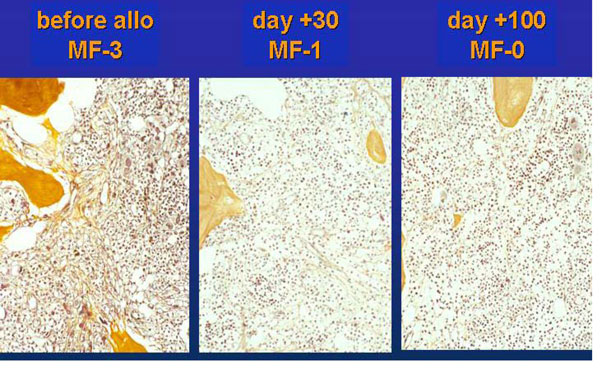
**Representative example of bone marrow regression in a patient after dose-reduced allografting on day +30 and +100**.
